# Targeting the PI3K/Akt/NF‐κB axis: Cluster of differentiation 5‐like‐mediated immunometabolic regulation of macrophage polarization in abdominal aortic aneurysm

**DOI:** 10.1002/ccs3.70048

**Published:** 2025-10-01

**Authors:** Hemoren Yi, Nan Liu, Zhengyang Wu, Lei Li, Tingting Li, Qixiang Liu, Man Duan, Taihu Wan

**Affiliations:** ^1^ Department of Vascular Surgery Qian Wei Hospital of Jilin Province Changchun China; ^2^ Department of General Surgery Qian Wei Hospital of Jilin Province Changchun China; ^3^ Department of Anesthesiology The First Hospital of Jilin University Changchun Jilin China; ^4^ College of Clinical Medicine Changchun University of Chinese Medicine Changchun Jilin China; ^5^ Department of Ultrasound Qian Wei Hospital of Jilin Province Changchun Jilin China; ^6^ Department of Vascular Surgery China‐Japan Union Hospital of Jilin University Changchun Jilin China; ^7^ Department of Interventional Oncology China‐Japan Union Hospital of Jilin University Changchun Jilin China

**Keywords:** abdominal aortic aneurysm, CD5L, macrophage polarization, NF‐κB, PI3K/Akt signaling pathway, RNA sequencing

## Abstract

Abdominal aortic aneurysm (AAA) is a life‐threatening vascular disorder lacking effective pharmacological interventions. We identified CD5 molecule‐like (CD5L) as a regulator of macrophage polarization in AAA via the phosphoinositide 3‐kinase/protein kinase B/nuclear factor kappa B (PI3K/Akt/NF‐κB) pathway. Transcriptomic analyses (GSE47472 and GSE57691) and angiotensin II (AngII)‐infused apolipoprotein E‐deficient (ApoE^−/−^) mice showed CD5L upregulation, inversely correlated with M1 macrophage infiltration. In vitro CD5L overexpression reduced, whereas knockdown increased M1 polarization and pro‐inflammatory cytokines in RAW264.7 cells and human monocyte‐derived macrophages. In vivo, CD5L knockdown aggravated aortic dilation, vascular disruption, and inflammatory mediator expression. Pharmacological modulation confirmed PI3K/Akt as essential for CD5L's anti‐inflammatory action: LY294002 amplified, whereas PI3K activator 740Y‐P mitigated CD5L deficiency effects. RNA sequencing confirmed PI3K/Akt activation downstream of CD5L. These results define CD5L as an immunometabolic checkpoint that suppresses NF‐κB‐mediated inflammation, suggesting a therapeutic target for AAA.

## Graphical abstract

Targeting the PI3K/Akt/NF‐κB Axis: CD5L suppresses M1 macrophage polarization and vascular inflammation, thereby attenuating abdominal aortic aneurysm progression.

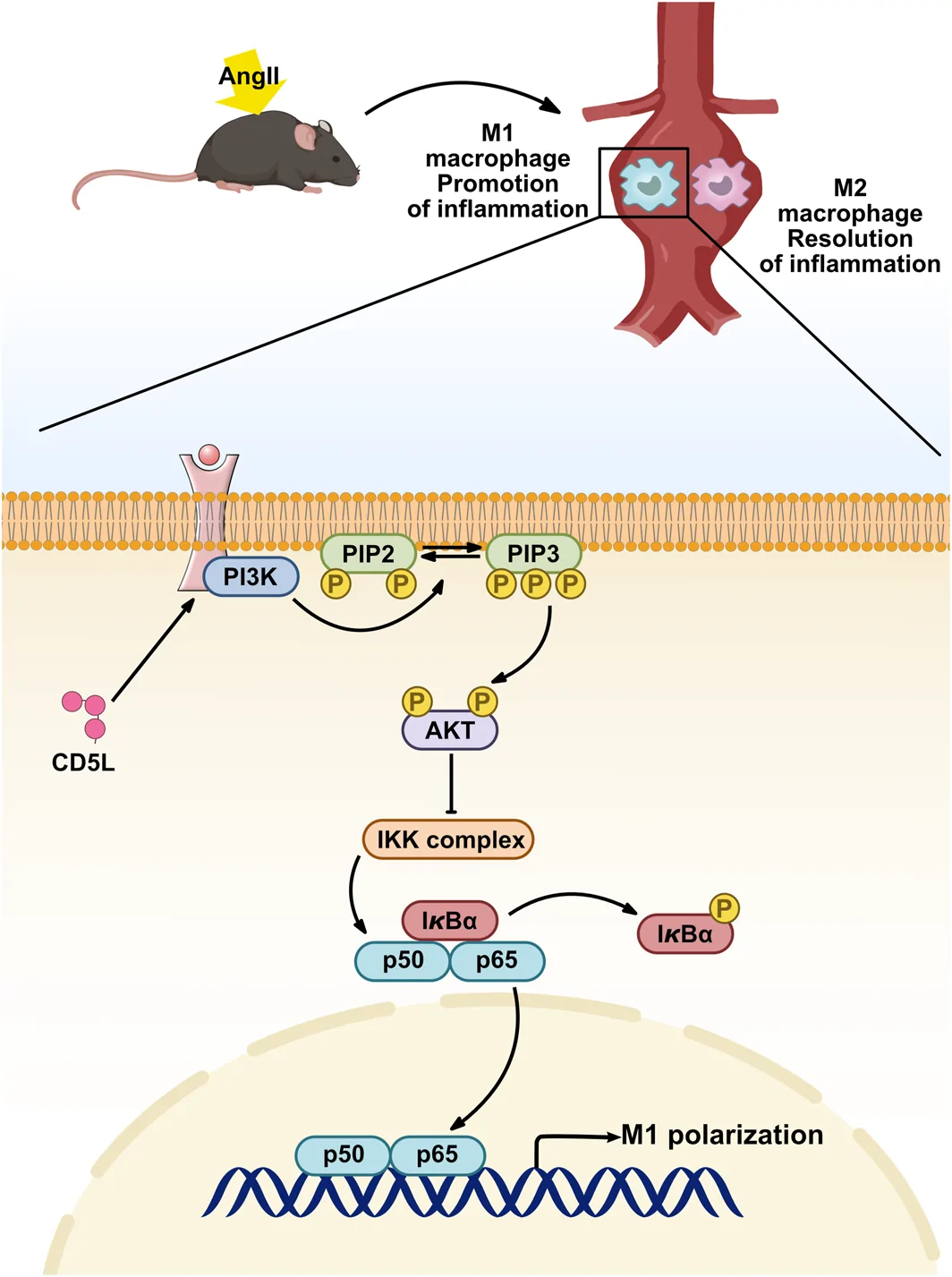

## 1 BACKGROUND

Abdominal aortic aneurysm (AAA) is a common and life‐threatening cardiovascular disease characterized by localized dilatation of the abdominal aortic wall.^1^ Its pathogenesis is complex, with infiltration and activation of inflammatory cells as a central mechanism.^2^ Elevated expression of adhesion molecules promotes monocyte/macrophage recruitment and activation, leading to the release of multiple inflammatory mediators that drive disease progression.^3^ Excessive expression of matrix metalloproteinases (MMPs) accelerates extracellular matrix (ECM) degradation, compromising vascular wall integrity,^4^, ^5^ whereas vascular smooth muscle cell apoptosis further disrupts vessel architecture.^6^, ^7^ Currently, no pharmacological agent effectively prevents or reverses AAA, leaving surgical intervention as the standard treatment. Elucidating the molecular mechanisms and identifying therapeutic targets are of high clinical importance.

Macrophage polarization has emerged as a key determinant of AAA progression. Proinflammatory M1 macrophages intensify vascular injury,^8^, ^9^ whereas M2 macrophages mediate anti‐inflammatory repair processes.^10^ Therapeutic modulation of macrophage phenotype is increasingly recognized as a promising avenue,^11^ with the PI3K/Akt/NF‐κB signaling pathway recognized as a key regulatory axis governing macrophage phenotype and inflammatory responses.^12^, ^13^

Cluster of differentiation 5‐like (CD5L), a soluble member of the scavenger receptor cysteine‐rich superfamily, is primarily secreted by macrophages.^14^ It exerts broad effects on lipid metabolism and immune regulation and has been implicated in diverse cardiovascular conditions.^15^, ^16^ CD5L has been shown to promote M2 polarization by inhibiting NF‐κB signaling, thereby exerting anti‐inflammatory and tissue‐repair functions.^17^, ^18^

Despite these insights, the role of CD5L in AAA remains insufficiently defined. In an Ang II‐induced ApoE^−/−^ mouse aneurysm model, administration of a neutralizing apoptosis inhibitor of macrophage (AIM)/CD5L antibody reduced aneurysm progression, decreased M1 macrophage proportion, and alleviated inflammation, yet its upstream/downstream signaling mechanisms were not delineated, and PI3K/Akt/NF‐κB pathway involvement was not assessed.^19^ Evidence from other inflammatory and cardiovascular contexts indicates that CD5L can regulate macrophage polarization through CD36‐mediated autophagy and restrain NF‐κB‐driven inflammation.^17^, ^18^ These findings suggest that CD5L may influence macrophage polarization and inflammatory signaling in AAA, potentially via the PI3K/Akt/NF‐κB axis. Moreover, although circulating CD5L has been linked to cardiovascular prognosis,^15^ its tissue distribution, cellular sources, and pathway‐specific roles in AAA remain to be elucidated.

Here, we combined RNA sequencing, an ApoE^−/−^ mouse AAA model, and multiple molecular assays to systematically characterize CD5L expression in AAA, assess its effects on macrophage polarization, and determine the involvement of the PI3K/Akt/NF‐κB pathway. This study aims to provide new mechanistic insights into inflammatory regulation in AAA and to lay the groundwork for developing CD5L‐targeted therapeutic strategies.

## 2 MATERIALS AND METHODS

### 2.1 Data collection and processing from the GEO database

Transcriptomic datasets GSE197748, GSE47472, and GSE57691 were retrieved from the gene expression omnibus (GEO). GSE197748 contained expression data from 11 murine AAA samples and 8 controls. The GSE47472 dataset included mRNA and long noncoding RNA expression profiles from 14 human AAA tissues and 8 donor abdominal aortic tissues, whereas the GSE57691 dataset included corresponding profiles from 49 human AAA tissues and 10 donor abdominal aortic tissues. As both GSE47472 and GSE57691 were generated on the GPL10558 platform, gene annotation was performed based on the platform's reference files prior to merging. To minimize potential batch effects across datasets, the Combat package in R (v4.2.2) was applied before integration. Differential expression of CD5L between the AAA and control groups was then assessed in both the merged dataset and the GSE197748 dataset using the Wilcoxon rank‐sum test.

### 2.2 Quantitative analysis of immune cell infiltration levels

Immune cell infiltration was quantified using the CIBERSORT algorithm applied to the merged expression profiles of the GSE47472 and GSE57691 datasets. Before analysis, expression data were normalized to ensure cross‐sample comparability. CIBERSORT was then used to estimate the relative proportions of 22 immune cell subsets per sample, with proportions standardized to sum to one. This approach enabled precise quantification and comparison of immune cell infiltration patterns between the AAA and control groups.

### 2.3 Correlation analysis between CD5L and immune cell infiltration abundance

Spearman's rank correlation analysis was performed on the merged GSE47472 and GSE57691 datasets to examine the associations between CD5L expression and immune infiltration. Relative abundances of 22 immune cell subsets were derived from CIBERSORT output, and correlation coefficients were calculated for each cell type to identify immune populations potentially influenced by CD5L within the AAA microenvironment.

### 2.4 Mouse grouping and treatment method

Male ApoE^−/−^ mice (C57BL/6J background, 10 weeks old) were purchased from Beijing Vital River Laboratory Animal Technology Co. Ltd. and housed under specific pathogen‐free conditions with free access to standard chow and water. A total of 40 mice were used in this study. All animal experiments were approved by the Animal ethics committee of Harbin Scientia Biotechnology Corporation (approval no. HRBSCIEC20230310) and conducted in compliance with ethical guidelines. Animal handling complied with internationally recognized standards for animal welfare, with all efforts made to minimize discomfort and stress. Mice were randomly assigned to four groups (*n* = 10 per group) and treated under inhalation anesthesia as follows: (i) Control, subcutaneous infusion of saline via osmotic pump (Alzet) for 28 days; (ii) AngII, subcutaneous infusion of angiotensin II (AngII, 1000 ng/kg/min; MCE) via osmotic pump for 28 days to induce AAA; (iii) siSCR, AngII infusion with twice‐weekly rapid tail vein injections of scrambled siRNA (non‐targeting sequence; RiboBio); and (iv) siCD5L, AngII infusion with twice‐weekly rapid tail vein injections of CD5L siRNA (target sequence 5′‐GCTCAATGTGAGCTAAATT‐3′; RiboBio).

### 2.5 Doppler ultrasound imaging

On day 14, the abdominal aortic diameter was evaluated using the Vevo 2100 imaging system (FUJIFILM VisualSonics). Mice were anesthetized with 2% isoflurane and positioned supine. After abdominal depilation, ultrasound transmission gel (Aqua Sonic, Parker Laboratories, NC9861677) was applied, and B‐mode imaging was performed along the longitudinal axis of the suprarenal aorta to measure the maximum inner diameter.

### 2.6 Histological staining and molecular analyses

Aortic tissues and surrounding structures were collected, fixed in 4% paraformaldehyde, embedded in paraffin, and sectioned at 5–10 μm. Sections were baked at 65°C for 2 h, deparaffinized in xylene (2 × 10 min), rehydrated through graded ethanol (100%, 95%, 80%), and rinsed in deionized water. All subsequent staining procedures were performed following this initial preparation.

#### 2.6.1 Hematoxylin and eosin (H&E) staining

Sections were stained with hematoxylin (3 min), differentiated in acid alcohol (15 s), blued (15 s), counterstained with eosin (3 min), dehydrated, cleared, and mounted with neutral resin. Slides were examined microscopically.

#### 2.6.2 Immunohistochemistry (IHC)

Following antigen retrieval (gastrin retrieval buffer, 37°C, 30 min) and quenching of endogenous peroxidase (3% H_2_O_2_, 10 min), sections were blocked with 5% bovine serum albumin (BSA) (30 min) and incubated overnight at 4°C with primary antibodies against CD5L (1:500, Abcam ab45408), TNFα (1:1000, Abcam ab34674), iNOS (1:2000, Abcam ab283655), p‐Akt S473 (1:100, CST #4060), or p‐NF‐κB p65 S536 (1:500, Abcam ab86299). After PBS washes, sections were incubated with goat anti‐rabbit secondary antibody (1:1000, Abcam ab6721) at 37°C for 30 min. 3,3′‐Diaminobenzidine substrate and the VECTASTAIN Elite ABC kit (VECTOR) were used for chromogenic detection (∼1 min), followed by hematoxylin counterstaining. Images were acquired with a BX53 microscope (Olympus, Japan) and analyzed using ImageJ to calculate mean optical density.

#### 2.6.3 Immunofluorescence

After blocking, sections were incubated overnight at 4°C with antibodies against iNOS (1:50, Abcam ab283655) or F4/80 (1:50, Abcam ab300421), washed, and incubated at 37°C for 1 h in the dark with Alexa Fluor 488 or 594 goat anti‐rabbit IgG (1:500, Abcam ab150077/ab150080). Nuclei were counterstained with DAPI (10 min). RAW264.7 cells were fixed with 3.7% paraformaldehyde, permeabilized with 0.1% Triton X‐100, blocked with 2% BSA/PBS, and incubated overnight with p‐NF‐κB p65 S536 (1:1000, CST #3033), followed by Alexa Fluor 488 secondary antibody (1:500, Abcam ab150077) for 1.5 h in the dark and DAPI staining. Fluorescence images were acquired using an FV3000 confocal microscope and quantified with ImageJ.

#### 2.6.4 Quantitative real‐time PCR

Total RNA was extracted using RNAiso Plus (TAKARA) and reverse‐transcribed with the PrimeScript RT kit with gDNA Eraser (TAKARA). Quantitative real‐time PCR (qRT‐PCR) was performed on a CFX96 Touch system (Bio‐Rad) using TB Green Premix Ex Taq II (TAKARA). β‐actin served as an internal reference, and relative expression was calculated using the 2^−ΔΔCt^ method. Primers were synthesized by GeneCreate Biotech, with sequences provided in Table 1.

**TABLE 1:** The primer sequences used in the assay.

Primer name	Nucleotide sequence
CD5L‐mouse‐F	GTTGGATCGTGTTTTTCAGA
CD5L‐mouse‐R	TCCCACTAGCTGCACTTTGGT
iNOS‐mouse‐F	TTGTGCGAAGTGTCAGTGG
iNOS‐mouse‐R	TCCTTTGAGCCCTTTGTGC
IL‐1β‐mouse‐F	TGCCACCTTTTGACAGTGATG
IL‐1β‐mouse‐R	AAGGTCCACGGGAAAGACAC
IL‐6‐mouse‐F	AGAGGAGACTTCACAGAGGATAC
IL‐6‐mouse‐R	TCATTTCCACGATTTCCCAGAG
β‐actin—mouse‐F	CACTGTCGAGTCGCGTCC
β‐actin—mouse‐R	TCATCCATGGCGAACTGGTG

### 2.7 Western blot analysis

Nuclear and cytoplasmic fractions of RAW264.7 cells were prepared using the NE‐PER Nuclear and Cytoplasmic Extraction Kit (Thermo, 78833), with cytoplasmic proteins obtained in hypotonic lysis buffer and nuclear proteins in hypertonic lysis buffer. Tissue lysates were generated using cell lysis buffer (Millipore). Equal amounts of protein were resolved by sodium dodecyl sulfate–polyacrylamide gel electrophoresis and transferred to polyvinylidene difluoride membranes (Beyotime, FFP39). Membranes were incubated with primary antibodies against CD5L (1:1000, Abcam ab45408), ICAM‐1 (1:1000, Abcam ab222736), VCAM‐1 (1:2000, Abcam ab134047), MMP‐2 (1:2000, Abcam ab92536), MMP‐9 (1:1000, Abcam ab38898), SM22α (1:1000, Abcam ab14106), elastin (1:200, Abcam ab217356), TNFα (1:1000, Abcam ab34674), iNOS (1:1000, Abcam ab283655), CD86 (1:1000, Abcam ab112490), p‐PI3K p85α (Tyr607) (1:500, Abcam ab182651), p‐Akt (Ser473) (1:2000, CST #4060), p‐IκBα (Ser36) (1:10000, Abcam ab133462), p‐NF‐κB p65 (Ser536) (1:2000, Abcam ab86299), histone H3 (1:1000, Abcam ab1791), and β‐actin (1:1000, Abcam ab8227). After phosphate‐buffered saline (PBS) washes, membranes were incubated with HRP‐conjugated goat anti‐rabbit IgG (1:2000, Abcam ab6721), and signals were detected by enhanced chemiluminescence. Band intensities were quantified using ImageJ software.

### 2.8 Cell sources and culture conditions

The murine macrophage‐like cell line RAW264.7 (derived from BALB/c mice) was authenticated by short tandem repeat profiling and confirmed mycoplasma‐free by PCR. Cells were cultured in high‐glucose Dulbecco's modified Eagle's medium (DMEM; Gibco, 8121728) enriched with 10% fetal bovine serum (fetal bovine serum (FBS); ExCell Bio), 100 U/mL penicillin, and 0.1 mg/mL streptomycin (Solarbio) at 37°C in a humidified 5% CO_2_ incubator.

Primary human monocyte‐derived macrophages (hMDMs) were obtained from peripheral blood mononuclear cells (PBMCs) isolated from healthy adult donors with informed consent. PBMCs were separated from EDTA‐anticoagulated whole blood using Ficoll‐Paque PLUS (GE Healthcare, #17‐1440‐03), and CD14^+^ monocytes were purified with magnetic microbeads (Miltenyi Biotec, #130‐050‐201). Monocytes were seeded at 1 × 10^6^ cells/well in six‐well plates and cultured in RPMI‐1640 medium (Gibco, #11875093) supplemented with 10% FBS, 1% penicillin–streptomycin, and 50 ng/mL macrophage colony‐stimulating factor (M‐CSF; PeproTech, #300‐25). Medium was refreshed every 3 days. After 6 days, adherent macrophages were evaluated by western blotting for TNF‐α and iNOS, and by flow cytometry for CD68, CD86, and CD206 to confirm M1 polarization status.

### 2.9 Gene silencing and stable cell line generation

For transient transfection, RAW264.7 cells were seeded at a density of 1.5 × 10^5^ cells/well in 6‐well plates and cultured in a 2 mL complete growth medium for 24 h to reach ∼50% confluence. CD5L siRNA or scrambled control siRNA (non‐targeting sequence; sequences provided in Table 2; RiboBio) at 110 pmol was diluted in 200 μL jetPRIME buffer (Polyplus, 114‐15), mixed with 4 μL jetPRIME reagent by vortexing, and incubated for 10 min at room temperature. The transfection mixture was added dropwise to the wells containing serum‐supplemented medium, gently swirled to ensure uniform distribution, and incubated at 37°C for 24 h.

**TABLE 2:** The siRNA sequences.

Name	Sequence (5′–3′)
si‐m‐cd5l_001	CAGAGTCTCCAACCAAAGT
si‐m‐cd5l_002	GTCGTGTTCTGGACAAGAA
si‐m‐cd5l_003	GCTCAATGTGAGCTAAATT

For stable overexpression, Lenti‐CD5L‐GFP and Lenti‐Vector constructs (GenePharma, Shanghai, China) were packaged in HEK293T cells. RAW264.7 cells at 70%–80% confluence were infected with viral supernatants at the appropriate multiplicity of infection calculated for each condition. After 48 h at 37°C, infected cells were selected with puromycin (Beyotime, ST551) until stable clones were established. CD5L expression levels were verified by western blotting. For hMDMs, the transfection procedure was similar to that of RAW264.7 cells. Differentiated human monocyte‐derived macrophages were seeded into 6‐well plates and transiently transfected with CD5L siRNA or scrambled control siRNA using the jetPRIME reagent, following the same transfection buffer composition and siRNA concentration. After 24 h of transfection, cells were collected for subsequent assays. Due to the poor compatibility of hMDMs with lentiviral transduction and antibiotic selection, only transient gene knockdown experiments were performed. CD5L overexpression and PI3K/Akt pathway modulation were not conducted in hMDMs.

### 2.10 Experimental grouping and treatments

RAW264.7 macrophages were randomly assigned to nine experimental groups (*n* = 3 independent biological replicates per group): (i) Control: cultured in complete DMEM without additional treatment; (ii) AngII: treated with 1 μM angiotensin II (MCE) for 24 h; (iii) siSCR: transfected with scrambled siRNA for 24 h, then exposed to AngII for 24 h; (iv) siCD5L: transfected with CD5L siRNA for 24 h, then exposed to AngII for 24 h; (v) Vector: stably transduced with Lenti‐Vector, followed by AngII for 24 h; (vi) CD5L: stably transduced with Lenti‐CD5L‐GFP, followed by AngII for 24 h; (vii) LY294002: pretreated with 10 μM LY294002 (PI3K/Akt inhibitor; MCE) for 1 h, followed by AngII for 24 h; (viii) 740Y‐P: pretreated with 50 μg/mL 740Y‐P (PI3K/Akt activator; MCE) for 1 h, followed by AngII for 24 h; and (ix) siCD5L + 740Y‐P: transfected with CD5L siRNA for 24 h, pretreated with 740Y‐P for 1 h, followed by AngII for 24 h. All chemical treatments were prepared in sterile PBS, and equivalent volumes of PBS were added to control groups as vehicle controls. The experimental grouping of hMDMs was similar to that of RAW264.7 cells, comprising four groups (*n* = 3 biological replicates per group): (i) Control: standard culture without treatment; (ii) AngII: treated with 1 μM angiotensin II for 24 h; (iii) siSCR: transfected with scrambled siRNA for 24 h followed by AngII stimulation for 24 h; (iv) siCD5L: transfected with CD5L siRNA for 24 h followed by AngII stimulation for 24 h. The transfection conditions and treatment durations were identical to those used for RAW264.7. CD5L overexpression and pharmacological pathway interventions were not applied in hMDMs.

### 2.11 Enzyme‐linked immunosorbent assay

Concentrations of TNF‐α and IL‐6 in cell culture supernatants and murine serum were determined using commercial enzyme‐linked immunosorbent assay (ELISA) kits (TNF‐α: ab208348; IL‐6: ab222503; Abcam) according to the manufacturer's protocols. Samples were centrifuged at 1000*g* for 10 min to remove debris, aliquoted, and stored at −80°C until analysis (no more than one freeze–thaw cycle). Each sample was measured in duplicate technical replicates, and absorbance was read at 450 nm using a microplate reader (BioTek Synergy H1). Concentrations were calculated from standard curves fitted using a four‐parameter logistic (4PL) model, and expressed as pg/mL.

### 2.12 Flow cytometry analysis

For basal phenotype detection, RAW264.7 cells (2 × 10^6^) were washed twice with ice‐cold PBS (Ca^2+^/Mg^2+^‐free, supplemented with 2% FBS) and stained with APC‐conjugated anti‐mouse CD206 (BioLegend, 141707; 0.2 mg/mL) and FITC‐conjugated anti‐mouse CD86 (Abcam, ab218757; 1:300) for 30 min at 4°C in the dark. Cells were then washed, resuspended in 300 μL PBS with 2% FBS, and analyzed on a NovoCyte flow cytometer (Agilent). At least 10,000 events were collected per sample, and data were processed with NovoExpress software (Agilent).

For M1 macrophage phenotype determination, cells were pre‐incubated with anti‐mouse CD16/CD32 (Fc block; Invitrogen, 14‐0161‐81; 1:1000) for 20 min at 4°C to minimize nonspecific binding, followed by staining with LIVE/DEAD Fixable Green viability dye (Invitrogen, L34965; 1:1000) and APC‐conjugated anti‐mouse CD86 antibody (Abcam, ab218757; 1:300) for 30 min at 4°C in the dark. After PBS washing, samples were acquired on the NovoCyte system, and M1 macrophage proportion and mean fluorescence intensity were quantified. Isotype and single‐color controls were included in each run for gating and compensation.

### 2.13 Mitochondrial respiration assay

Mitochondrial oxidative function was evaluated with the Seahorse XF Cell Mito Stress Test Kit (Agilent). RAW264.7 cells were seeded at 1.0 × 10^4^ cells/well in XF96 microplates and cultured for 24 h to allow attachment. Before assay, the growth medium was replaced with pre‐warmed bicarbonate‐free XF Base Medium supplemented with 10 mM glucose, 1 mM sodium pyruvate, and 2 mM L‐glutamine, and cells were equilibrated for 45 min at 37°C in a non‐CO_2_ incubator. Sequential injections of oligomycin (1 μM), FCCP (1 μM), and rotenone/antimycin A (0.5 μM each) were performed automatically by the Seahorse XF Analyzer (Agilent), with three oxygen consumption rate (OCR) measurements taken after each injection. Data were processed with Wave software (Agilent), normalized to protein content per well (bicinchoninic acid assay), and used to calculate basal, maximal, adenosine triphosphate (ATP)‐linked, and non‐mitochondrial respiration.

### 2.14 RNA sequencing and differential gene expression analysis

Total RNA was isolated from RAW264.7 cells in the Ang II and siCD5L groups (*n* = 3 per group) using Trizol reagent (Invitrogen) and treated with DNase I to eliminate genomic DNA. After rRNA depletion, cDNA libraries were generated and sequenced on the Illumina HiSeq 2500 platform (Illumina). Raw data underwent quality control, and differentially expressed genes (DEGs) were identified with the Limma package in R (v4.2.2) using thresholds of |log_2_ fold change| > 1.0 and *p* < 0.05. DEGs were visualized with the ggplot2 package (v3.4.0).

### 2.15 Functional enrichment analysis

Kyoto encyclopedia of genes and genomes (KEGG) pathway analysis of DEGs was performed with the clusterProfiler package in R. Significance was defined as *p* < 0.05 and false discovery rate (FDR) <0.1. Enrichment outcomes were visualized in R.

### 2.16 Gene set enrichment analysis

Gene set enrichment analysis (GSEA) was performed using RNA‐seq data and the GSEA software (version 3.0; Broad Institute). The “c2.cp.Reactome.v7.4.symbols” gene set collection was obtained from the Molecular Signatures Database. Gene set size was restricted to 5–5000 genes, and 1000 permutations were applied. Statistical significance was defined as *p <* 0.05.

### 2.17 Determination of LY294002 IC_50_

RAW264.7 cells were seeded in 96‐well plates and exposed to graded concentrations of LY294002 (MedChemExpress, HY‐10108) for 24 h.^20^ Cell viability was determined by the CCK‐8 assay (Dojindo). After adding 10 μL reagent per well and incubating at 37°C for 4 h, absorbance was measured at 450 nm with a microplate reader. IC_50_ values were calculated from triplicate experiments using GraphPad Prism 9.

### 2.18 Statistical analysis methods

All experiments were performed in at least three independent replicates to ensure reliability and reproducibility. Data normality was assessed using the Shapiro–Wilk test. For normally distributed data, comparisons were performed using a two‐tailed unpaired Student's *t*‐test; if variances were unequal, Welch's *t*‐test was applied. For non‐normally distributed data, a two‐tailed Mann–Whitney test was used. Statistical analyses were conducted using GraphPad Prism 9 (GraphPad Software), and *p* ≤ 0.05 was considered statistically significant. Sample sizes and exact *p* values are indicated in the figure legends to ensure transparency and reproducibility.

## 3 RESULTS

### 3.1 Significant upregulation of CD5L in AAA

**FIGURE 1:**
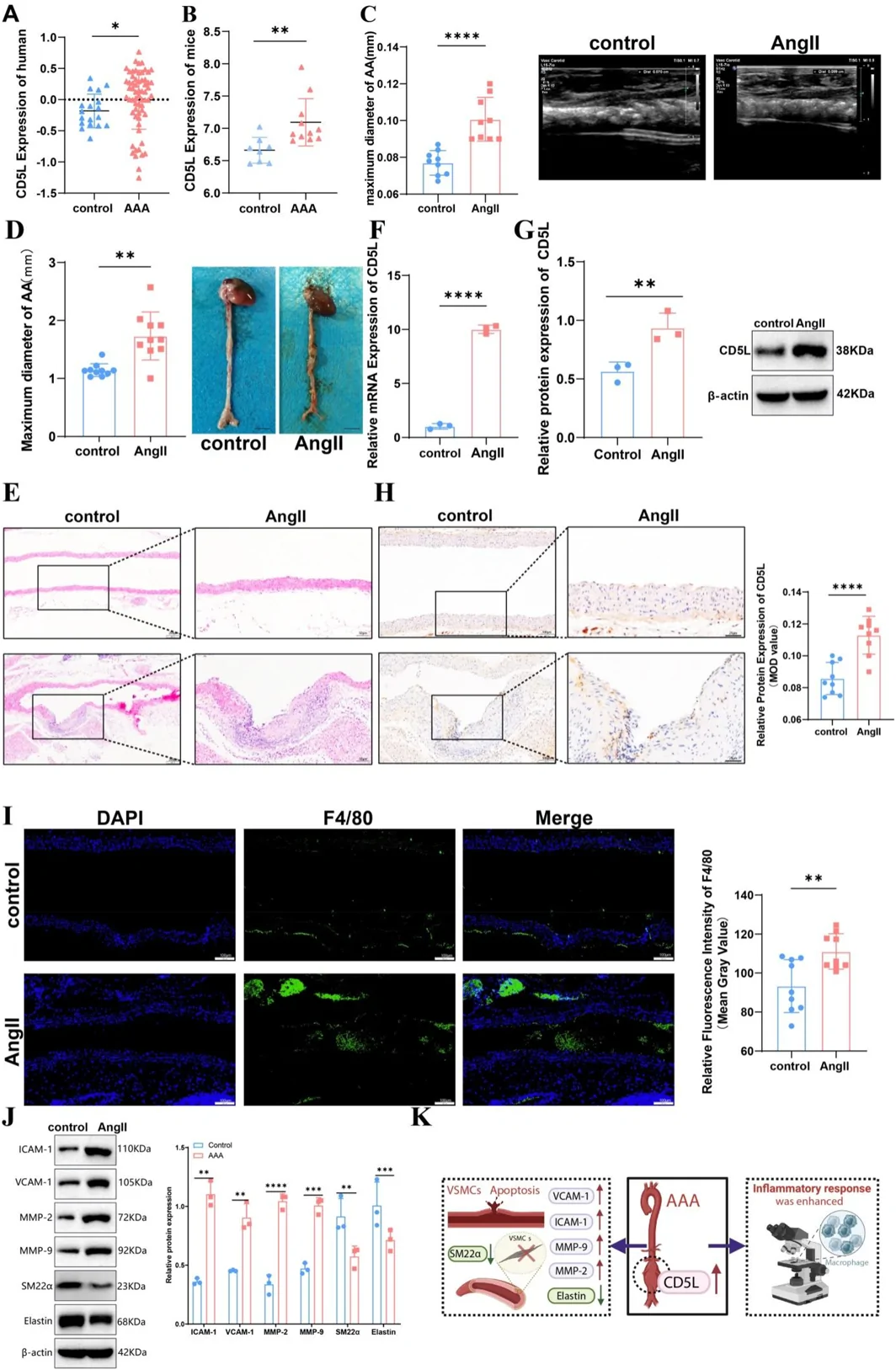
CD5L upregulation in AAA and associated pathological features. (A) CD5L expression in human (GSE47472 and GSE57691) and (B) murine (GSE197748) datasets. (C) Doppler ultrasound images and quantification of maximal inner abdominal aortic diameter at day 14 (*n* = 10). (D) Gross images and quantification of maximal outer diameter at day 28 (*n* = 10). (E) H&E‐stained sections of abdominal aorta (*n* = 10). (F) qRT‐PCR of CD5L mRNA levels (*n* = 3). (G) Western blot of CD5L protein with densitometry (*n* = 3). (H) Immunohistochemical staining of CD5L (*n* = 9). (I) Immunofluorescence staining of F4/80‐positive macrophages (*n* = 9). (J) Western blot of AAA pathological markers (*n* = 3). (K) Schematic of CD5L upregulation and potential roles in AAA pathogenesis. Representative images are shown. Sample sizes correspond to methods: *n* = 10, animals per group; *n* = 3, biological replicates for molecular assays; *n* = 9, samples for histological quantification. Scale bars, 5 mm (D), 200 μm (E), 100 μm (H and I). AAA, abdominal aortic aneurysm; CD5L, cluster of differentiation 5‐like; H&E, hematoxylin and eosin; qRT‐PCR, quantitative real‐time PCR. Data are mean ± SD; *p < 0.05; **p < 0.01; ***p < 0.001; ****p < 0.0001.

In human AAA datasets GSE47472 and GSE57691, CD5L expression was higher in AAA tissues compared with controls (*p* < 0.05) (Figure 1A). A similar increase was observed in the murine AAA dataset GSE197748 (*p <* 0.01) (Figure 1B). In the AngII‐induced ApoE^−/−^ mouse model,^21^ Doppler ultrasound revealed a marked increase in the abdominal aortic inner diameter at day 14 (*p <* 0.0001) and an increase in outer diameter at day 28 (*p <* 0.01) (Figure 1C,D). Histological analysis showed severe disruption of the aortic wall structure in AAA mice (Figure 1E).

qRT‐PCR, Western blot analysis, and IHC confirmed a marked upregulation of CD5L in the AngII group (Figure 1F–H). Immunofluorescence also revealed substantial macrophage accumulation within the lesions (Figure 1I). Moreover, expressions of VCAM‐1, ICAM‐1, MMP‐2, and MMP‐9 were increased, whereas elastin and SM22α were decreased in AAA tissues (Figure 1J). Collectively, these findings indicate that CD5L is consistently upregulated during AAA development and is associated with characteristic pathological changes (Figure 1K).

### 3.2 Study on the exacerbating effect of CD5L knockdown on AAA progression

**FIGURE 2:**
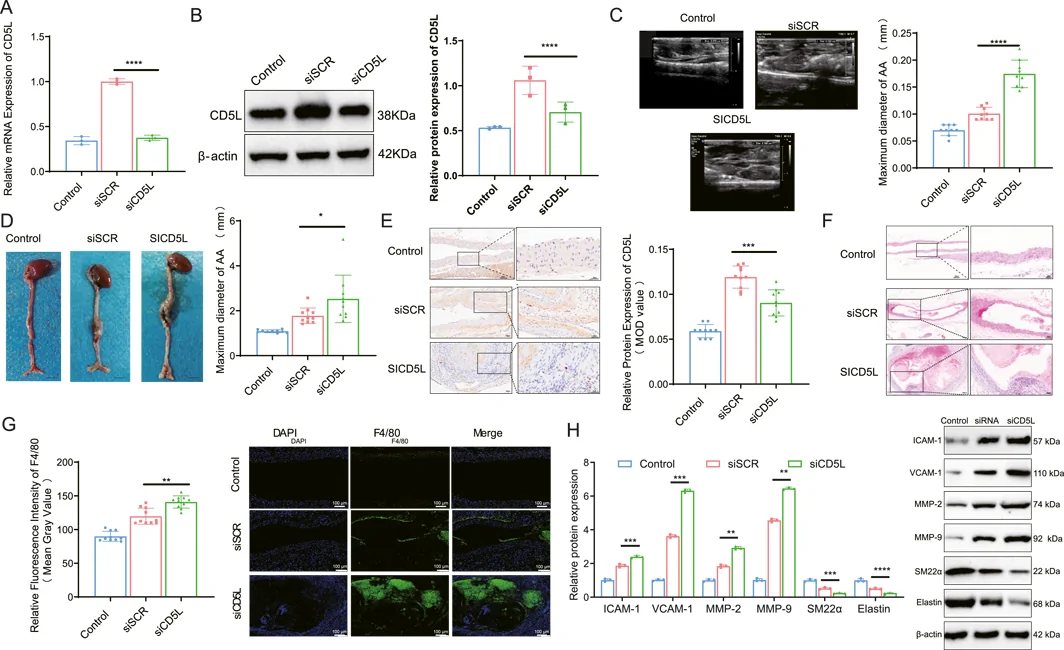
Effects of CD5L knockdown on AAA progression. (A) qRT‐PCR analysis of CD5L mRNA levels in the AAA mouse model after CD5L knockdown (*n* = 3). (B) Western blot and densitometric analysis of CD5L protein (*n* = 3). (C) Doppler ultrasound images and quantification of maximal inner aortic diameter at day 14 (*n* = 10). (D) Representative photographs and quantification of maximal outer aortic diameter at day 28 (*n* = 10). (E) Immunohistochemical staining of CD5L (*n* = 9). (F) H&E staining of abdominal aorta (*n* = 10). (G) Immunofluorescence staining of F4/80‐positive macrophages (*n* = 9). (H) Western blot and densitometric analysis of AAA‐related pathological markers (*n* = 3). Scale bars: (D), 5 mm; (E and G), 100 μm; (F), 200 μm. *n* values correspond to those described in the methods: *n* = 10, total animals per group; *n* = 3, biological replicates for molecular assays; *n* = 9, samples for quantitative histological analysis. AAA, abdominal aortic aneurysm; CD5L, cluster of differentiation 5‐like; H&E, hematoxylin and eosin; qRT‐PCR, quantitative real‐time PCR. Data are mean ± SD; *p < 0.05; **p < 0.01; ***p < 0.001; ****p < 0.0001. [Correction added on 20 September 2026, after first online publication: The Figure 2 has been updated in this version.]

Tail vein delivery of CD5L siRNA significantly reduced CD5L expression in mice, as confirmed by qRT‐PCR and western blotting (Figure 2A,B). Compared with the siSCR group, CD5L knockdown led to a greater increase in maximal aortic inner diameter at day 14 and maximal outer diameter at the endpoint of AngII treatment (Figure 2C,D). IHC analysis further verified markedly lower CD5L expression in the siCD5L group (Figure 2E). H&E staining revealed more severe aortic wall disruption and thrombus formation in the siCD5L group (Figure 2F). Immunofluorescence showed a higher accumulation of F4/80‐positive macrophages (Figure 2G). Western blot analysis indicated upregulation of VCAM‐1, ICAM‐1, MMP‐2, and MMP‐9, accompanied by decreased elastin and SM22α levels in the siCD5L group (Figure 2H).

### 3.3 CD5L slows AAA progression by inhibiting macrophage polarization to the M1 phenotype

**FIGURE 3:**
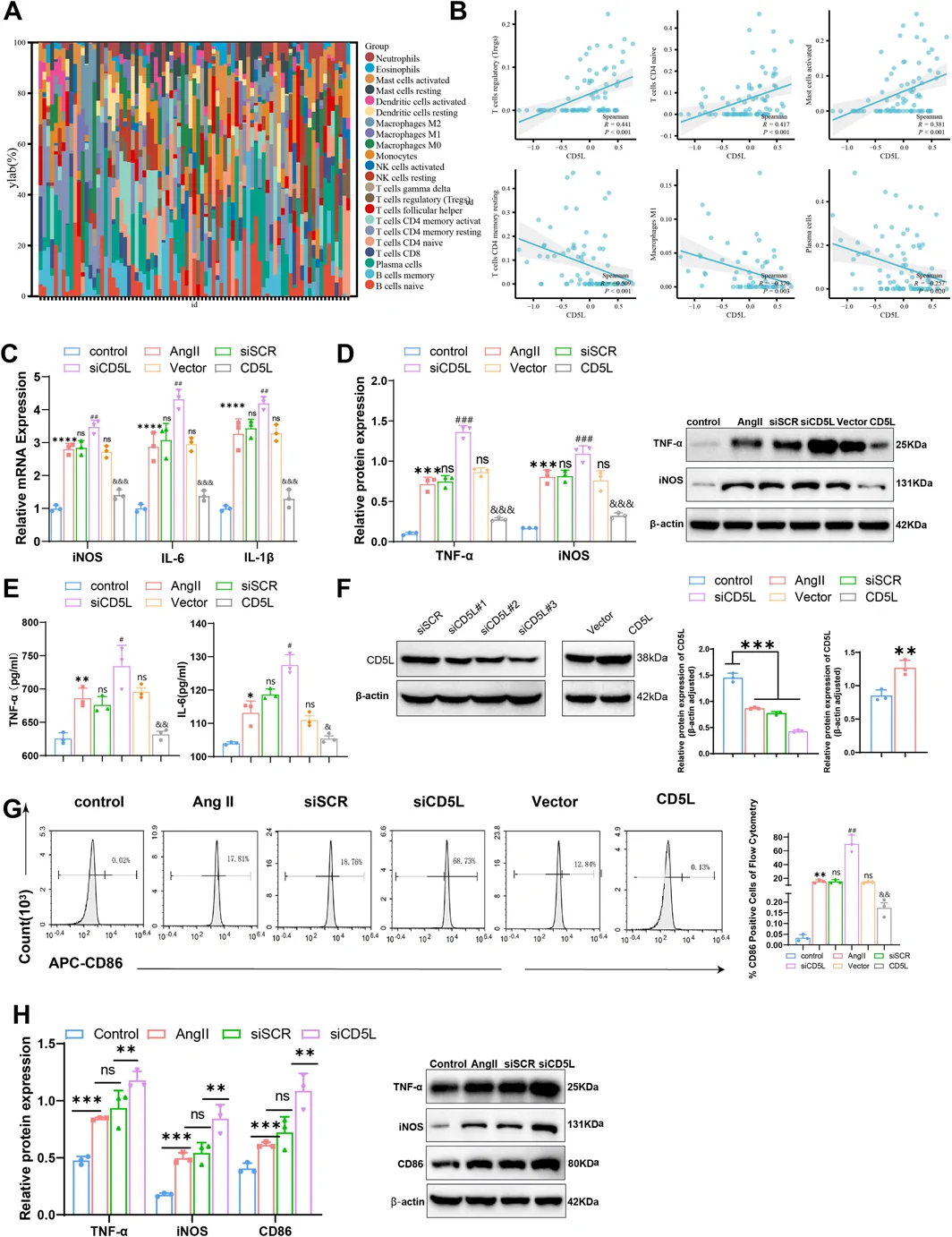
Effects of CD5L on macrophage M1 polarization in the AAA condition. (A) Heatmap showing the distribution of 22 immune cell subpopulations in control and AAA samples. (B) Histogram illustrating the correlation between CD5L expression and various infiltrating immune cells. (C) qRT‐PCR analysis of iNOS, IL‐6, and IL‐1β expressions in RAW264.7 cells (*n* = 3). (D) Western blot and densitometric quantification of TNF‐α and iNOS in RAW264.7 cells (*n* = 3). (E) ELISA quantification of TNF‐α and IL‐6 in the supernatant of RAW264.7 cell cultures (*n* = 3). (F) Western blot and densitometric analysis of CD5L knockdown and overexpression efficiency in RAW264.7 cells (*n* = 3). (G) Flow cytometry quantification and representative histograms showing M1 macrophages (CD86^+^) in RAW264.7 cells (*n* = 3). (H) Western blot and densitometric analysis of time‐dependent changes in CD5L, TNF‐α, and iNOS expressions in AngII‐treated RAW264.7 cells (*n* = 3). Data are presented as mean ± SD.; *n* values correspond to biological replicates. AAA, abdominal aortic aneurysm; CD5L, cluster of differentiation 5‐like; ELISA, enzyme‐linked immunosorbent assay; qRT‐PCR, quantitative real‐time PCR. Statistical significance is indicated as *p < 0.05; **p < 0.01; ***p < 0.001; ****p < 0.0001; ns denotes no significant difference. Significance symbols: * versus control; # versus siSCR; and versus vector; *p <* 0.0001 versus 0 h; ^##^ *p <* 0.01 versus 18 h; comparison vs. siSCR group ^###^ *p* < 0.001; ^####^ *p <* 0.0001 versus 18 h; comparison vs. Vector group ^&&^ *p* < 0.01, ^&&&^ *p* < 0.001. [Correction added on 20 September 2026, after first online publication: The Figure 3 has been updated in this version.]

CIBERSORT analysis of merged GSE47472 and GSE57691 datasets revealed altered immune cell distributions between AAA and control samples (Figure 3A). Spearman correlation revealed positive associations of CD5L expression with regulatory T cells, naïve CD4^+^ T cells, and activated mast cells while showing negative associations with resting memory CD4^+^ T cells, M1 macrophages, and plasma cells (Figure 3B).

In vitro flow cytometry confirmed that RAW264.7 cells exhibited an M0 macrophage phenotype (Figure S1A). AngII stimulation increased CD5L expression and enhanced M1 polarization in both RAW264.7 cells and hMDMs, as evidenced by increased iNOS, IL‐6, and IL‐1β expressions on qRT‐PCR and western blotting (Figure 3C,D; Figure S1B,C), and by elevated TNF‐α and IL‐6 levels in culture supernatants detected by ELISA (Figure 3E; Figure S1D). Flow cytometry also demonstrated a higher proportion of CD86‐positive cells after AngII treatment (Figure 3G).

Time‐course analysis in AngII‐treated RAW264.7 cells showed gradual increases in CD5L expression, whereas TNF‐α and iNOS peaked at 18 h and declined by 24 h but remained above the baseline (Figure 3H). CD5L knockdown via siRNA and overexpression via lentiviral transduction were confirmed by western blotting (Figure 3F; Figure S1E). Knockdown increased, whereas overexpression decreased M1 polarization in both RAW264.7 cells and hMDMs, as indicated by gene expression, protein levels, and cytokine secretion (Figure 3C–E,G; Figure S1B–D).

**FIGURE 4:**
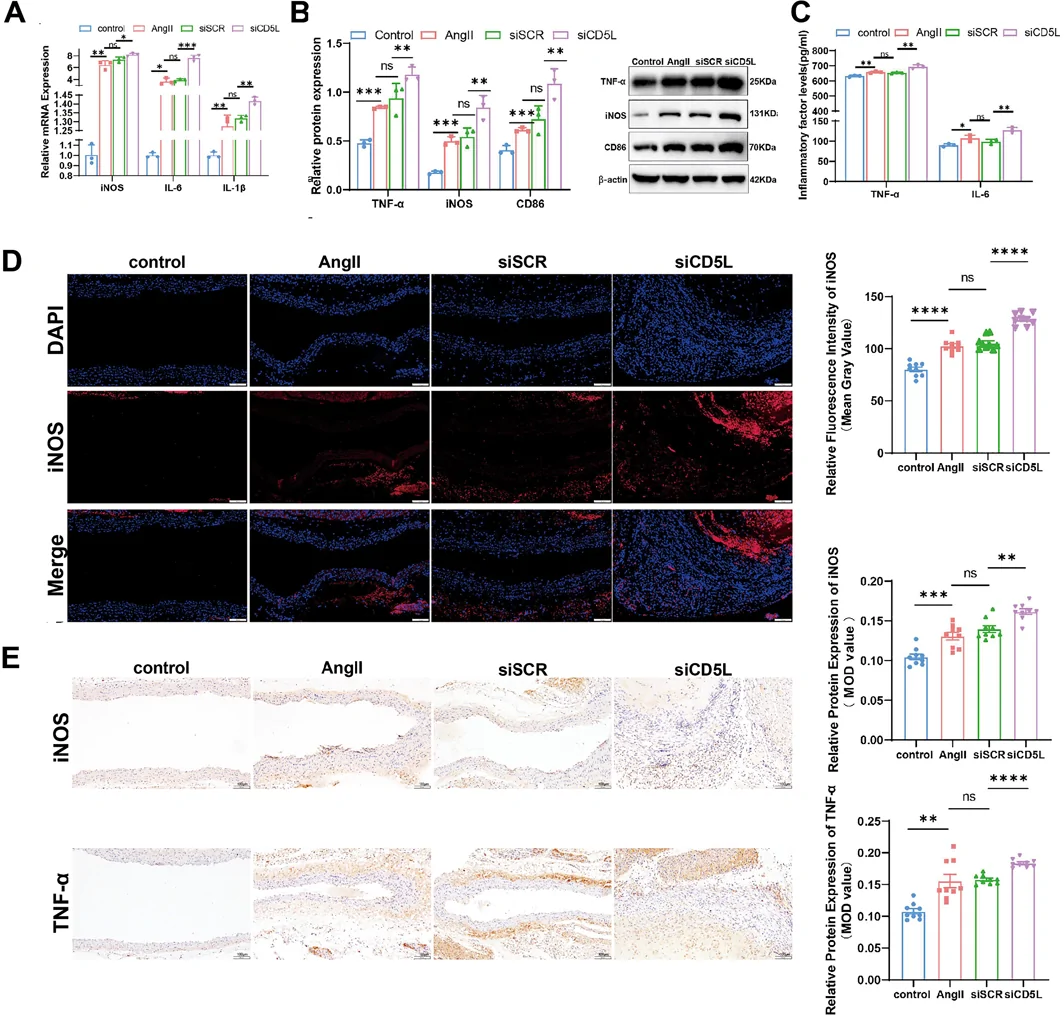
Effects of CD5L on macrophage M1 polarization in AAA. (A) qRT‐PCR analysis of iNOS, IL‐6, and IL‐1β expressions in murine abdominal aortic tissue (*n* = 3). (C) ELISA quantification of TNF‐α and IL‐6 in murine serum (*n* = 3). (B) Western blot and densitometric analysis of TNF‐α, iNOS and CD86 expression in murine abdominal aortic tissue (*n* = 3). (D) Representative immunofluorescence images and quantification of M1 macrophages (iNOS) in murine abdominal aortic tissue (*n* = 9; scale bar, 100 μm). (E) Representative IHC images and quantification of TNF‐α and iNOS in murine abdominal aortic tissue (*n* = 9; scale bar, 100 μm). Data are presented as mean ± SD; *n* values correspond to biological replicates. AAA, abdominal aortic aneurysm; CD5L, cluster of differentiation 5‐like; ELISA, enzyme‐linked immunosorbent assay; IHC, immunohistochemistry; qRT‐PCR, quantitative real‐time PCR. Statistical significance is indicated as *p < 0.05; **p < 0.01; ***p < 0.001; ****p < 0.0001; ^##^ *p* < 0.01; ^###^ *p* < 0.001; ^####^ *p* < 0.0001; ns denotes no significant difference.

In the ApoE^−/−^ mouse AAA model, CD5L knockdown increased iNOS, IL‐6, and IL‐1β mRNA (Figure 4A), elevated TNF‐α and iNOS protein levels (Figure 4B), and increased serum TNF‐α and IL‐6 levels (Figure 4C). Immunofluorescence and IHC showed greater accumulation of iNOS‐positive and TNF‐α‐positive macrophages in the abdominal aortic wall (Figure 4D,E).

### 3.4 Role of CD5L in reducing macrophage M1 polarization in AAA by activating the PI3K/Akt signaling pathway

**FIGURE 5:**
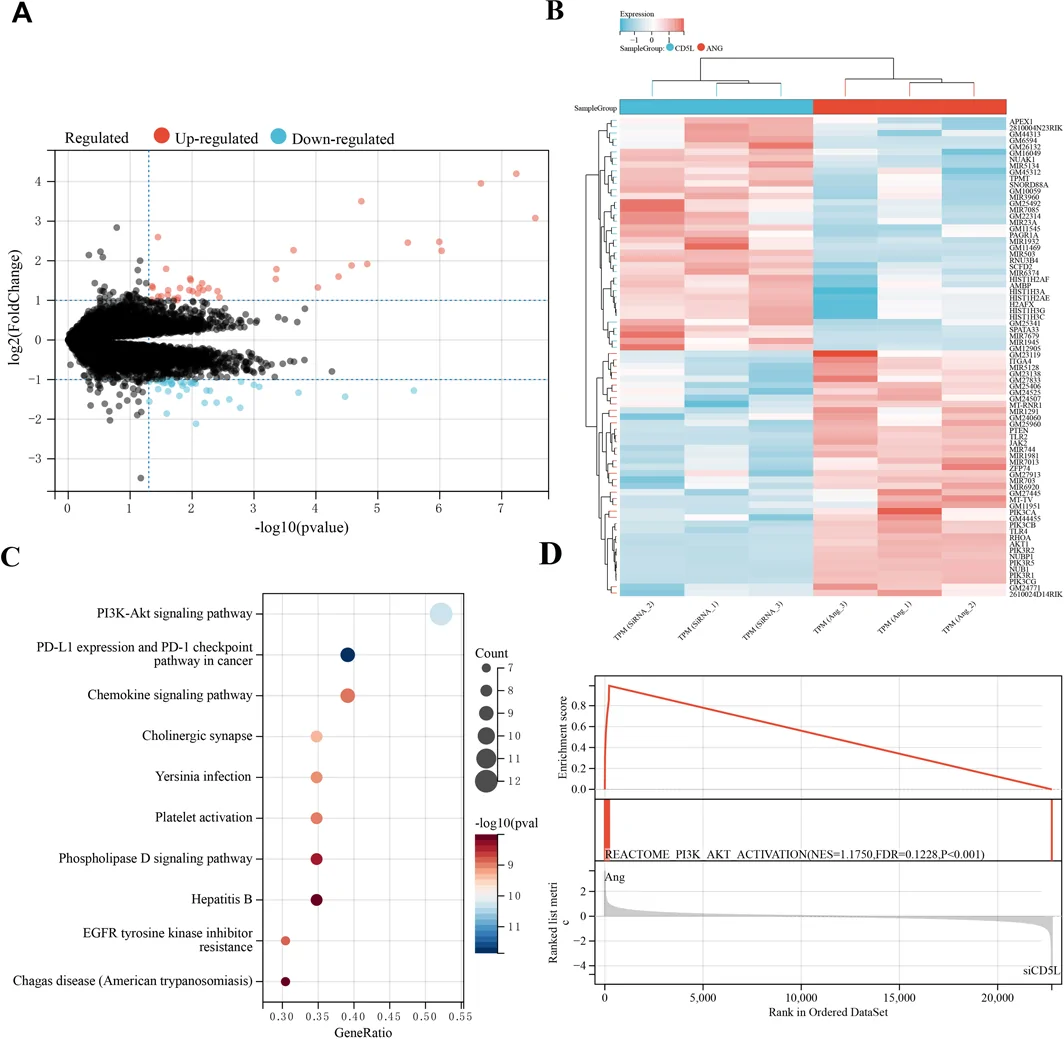
RNA‐seq and bioinformatics analysis reveal that CD5L inhibits M1 polarization through activation of the PI3K/Akt signaling pathway. (A) Volcano plot depicting the log_2_ fold change versus −log_10_ *p* values for DEGs between Ang II‐treated and siCD5L‐transfected RAW264.7 cells, with upregulated genes shown in red and downregulated genes shown in blue. (B) Heatmap illustrating the relative expression patterns of DEGs between the two groups. (C) Bubble plot of the top 10 significantly enriched KEGG pathways, with the PI3K/Akt signaling pathway highlighted. Significance thresholds: *p* value < 0.05 and FDR < 0.1. (D) Representative GSEA enrichment plot demonstrating significant association of the RNA‐seq gene expression profile with the REACTOME_PI3K_AKT_ACTIVATION pathway. CD5L, cluster of differentiation 5‐like; DEGs, differentially expressed genes; FDR, false discovery rate; GSEA, gene set enrichment analysis. Significance thresholds: |NES| ≥ 1.0, nominal *p* < 0.05, and FDR < 0.25.

RNA‐seq of AngII‐treated and siCD5L RAW264.7 cells identified 76 DEGs (39 upregulated and 37 downregulated; |log_2_ fold‐change| > 1.0, *p <* 0.05; Table 3), as shown in the volcano plot and heatmap (Figure 5A,B). KEGG analysis highlighted the PI3K/Akt pathway among the top 10 enriched pathways (*p <* 0.05, FDR < 0.1; Figure 5C). GSEA further confirmed positive enrichment of the REACTOME_PI3K_AKT_ACTIVATION pathway (|NES| ≥ 1.0, nominal *p <* 0.05, FDR < 0.25; Figure 5D). In addition, mitochondrial stress analysis showed that AngII markedly impaired aortic mitochondrial respiration, with reduced basal OCR and ATP production (Figure S1F).

**TABLE 3:** Expression levels of DEGs.

DEGs	logFC	*p* value
SNORD88A	−2.11693529	0.008627108
MIR3960	−1.858799652	0.025695179
MIR6374	−1.714171614	0.001671147
MIR7679	−1.589482723	0.006263572
GM11545	−1.583086896	0.004016412
MIR23A	−1.55488622	0.048829814
MIR1932	−1.500318353	0.00250206
RNU3B4	−1.432155733	3.38E‐05
MIR7085	−1.412077047	0.015991291
NUAK1	−1.332057733	0.000191032
GM44313	−1.296963272	0.02438923
MIR1945	−1.282306662	0.00513762
MIR503	−1.279009046	2.62E‐06
TPMT	−1.27483271	0.005838397
GM16049	−1.25143215	0.012182568
HIST1H3C	−1.242735259	0.035259281
MIR5134	−1.183806012	0.000819026
GM25341	−1.157952403	0.014275636
SPATA33	−1.131186026	0.00100485
GM26132	−1.122335618	0.021387631
APEX1	−1.108249083	0.021307101
HIST1H2AF	−1.101591245	0.010276794
GM12905	−1.101477266	0.008768735
PAGR1A	−1.092953769	0.020958416
GM25492	−1.091957671	0.013670442
2810004N23RIK	−1.090871579	0.028738151
H2AFX	−1.079857391	0.02666923
GM11469	−1.079116164	0.009590158
GM6594	−1.067377393	0.010836107
HIST1H2AE	−1.063186554	0.015308987
SCFD2	−1.055986941	0.001615114
GM45312	−1.052114366	0.049152659
HIST1H3G	−1.046946447	0.03210599
AMBP	−1.033115559	0.022960554
HIST1H3A	−1.029482742	0.037545974
GM10059	−1.015722934	0.021880823
GM22314	−1.009885847	0.027969791
MIR1291	1.007671491	0.017344314
GM11951	1.016032934	0.044652092
PIK3CA	1.037606732	0.019385431
GM24507	1.067533259	0.016851322
GM25406	1.07493835	0.003606504
GM44455	1.094666238	0.035191593
GM27833	1.103501352	0.031331567
GM27445	1.138348958	0.024629227
MIR6920	1.176702435	0.008070889
GM24771	1.229699595	0.009787711
ITGA4	1.232849242	0.003875005
GM24525	1.255387735	0.024355768
2610024D14RIK	1.256357951	0.011096409
MIR5128	1.258645885	0.006641668
MIR7013	1.306533478	0.005421674
GM23138	1.31005519	0.015639704
MIR703	1.320393511	0.007641218
GM23119	1.320556234	0.043159426
JAK2	1.324911758	9.30E‐05
GM24060	1.35649674	0.044354249
GM25960	1.433767256	0.006887471
ZFP74	1.509727198	0.010527409
MIR1981	1.53826432	0.000444595
MT‐TV	1.548817224	0.010740345
PIK3CB	1.600702818	4.31E‐05
MIR744	1.787802973	0.00043399
MT‐RNR1	1.796023397	0.026255345
AKT1	1.879627224	2.65E‐05
PTEN	1.917890885	1.48E‐05
PIK3R2	2.252364413	9.33E‐07
TLR4	2.265487024	0.000229692
RHOA	2.457287556	3.28E‐06
NUBP1	2.479004339	1.02E‐06
GM27913	2.596981931	0.035587623
PIK3R5	3.077690058	2.86E‐08
TLR2	3.50320629	1.84E‐05
PIK3CG	3.954148662	2.16E‐07
NUB1	4.197548137	5.81E‐08
PIK3R1	4.454212152	5.21E‐08

Abbreviation: DEGs, differentially expressed genes.

### 3.5 CD5L regulates the PI3K/Akt/NF‐κB axis in the AAA microenvironment

**FIGURE 6:**
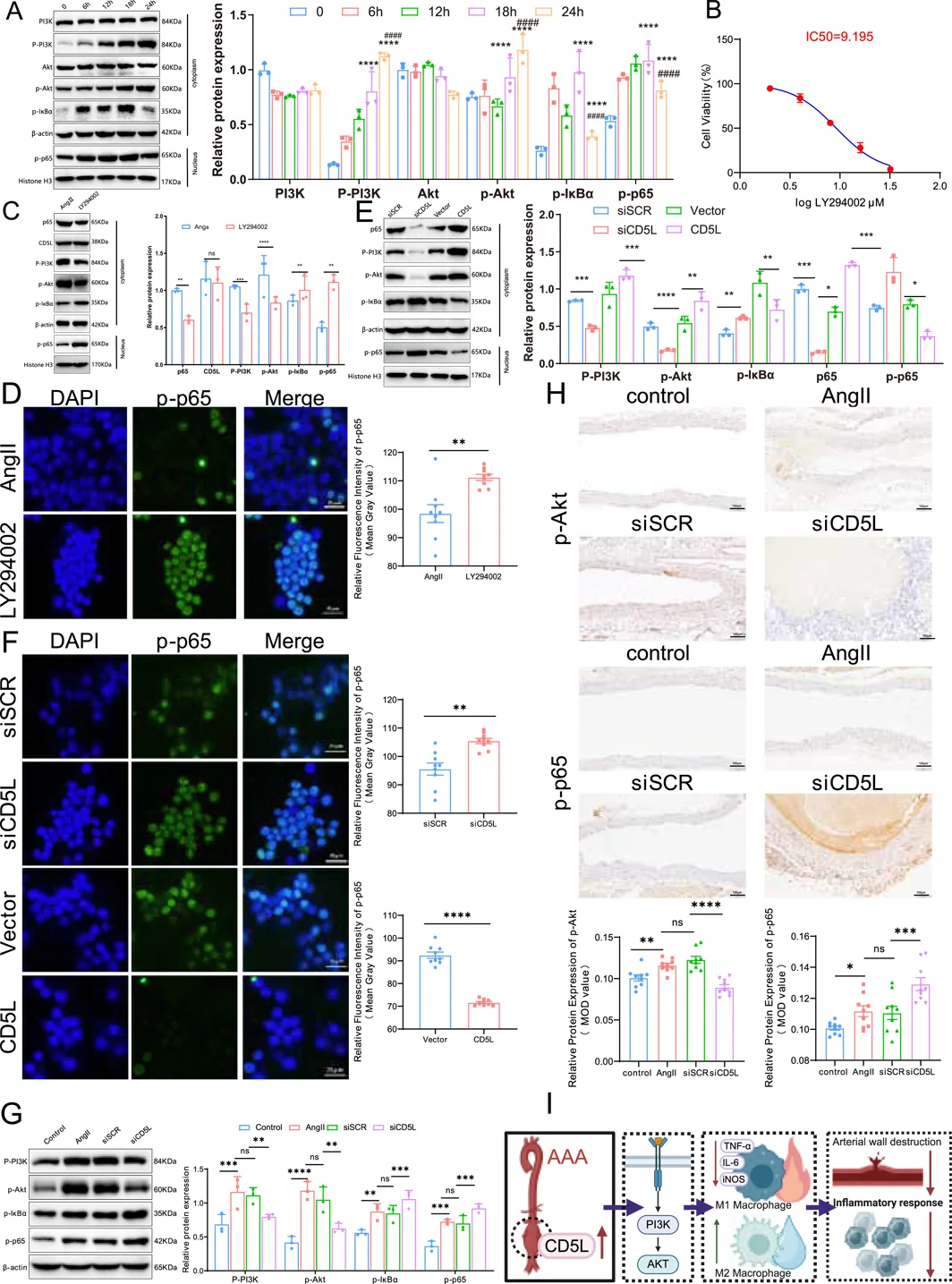
CD5L regulates the PI3K/Akt/NF‐κB axis in the AAA microenvironment. (A) Western blot analysis showing time‐dependent changes in phosphorylated PI3K, Akt, IκBα, and p65 in Ang II‐treated RAW264.7 cells, with densitometric quantification (*n* = 3). (B) CCK‐8 assay‐based dose–response curve showing the viability of RAW264.7 cells exposed to increasing concentrations of the PI3K inhibitor LY294002 for 24 h. (C) Western blot analysis of CD5L and PI3K/Akt/NF‐κB‐related proteins in RAW264.7 cells (*n* = 3). (D) Representative immunofluorescence images and quantification of phosphorylated p65 (green) in RAW264.7 cells, counterstained with DAPI (blue); scale bar, 25 μm (*n* = 9). (E) Western blot analysis of PI3K/Akt/NF‐κB‐related proteins in RAW264.7 cells under the indicated treatments (*n* = 3). (F) Immunofluorescence analysis of phosphorylated p65 in RAW264.7 cells; scale bar, 25 μm (*n* = 9). (G) Western blot analysis of PI3K/Akt/NF‐κB‐related proteins in murine abdominal aortic tissue (*n* = 3). (H) Immunohistochemistry of phosphorylated Akt and p65 in murine abdominal aortic tissue; scale bar, 100 μm (*n* = 9). (I) Schematic diagram illustrating the proposed mechanism by which CD5L activates the PI3K/Akt signaling pathway to suppress macrophage M1 polarization in AAA. Data are mean ± SD. AAA, abdominal aortic aneurysm. AAA, abdominal aortic aneurysm; CD5L, cluster of differentiation 5‐like. Statistical significance: *p < 0.05; **p < 0.01; ***p < 0.001; ****p < 0.0001; ns, not significant; ***p < 0.0001 versus 0 h; ^####^ *p <* 0.0001 versus 18 h. [Correction added on 20 September 2026, after first online publication: The Figure 6 has been updated in this version.]

Time‐course western blot analysis of AngII‐treated RAW264.7 macrophages revealed a progressive increase in cytoplasmic p‐PI3K and p‐Akt, as well as p‐IκBα and nuclear p‐p65, peaking at 18 h and remaining significantly elevated at 24 h compared with baseline (Figure 6A). Treatment with the PI3K inhibitor LY294002 (10 μM, selected based on CCK‐8 viability assays; Figure 6B) markedly reversed AngII‐induced phosphorylation of PI3K, Akt, IκBα, and p65 (Figure 6C), and enhanced p‐p65 nuclear translocation (Figure 6D). CD5L knockdown reduced p‐PI3K and p‐Akt, increased p‐IκBα, and promoted nuclear accumulation of p‐p65, whereas CD5L overexpression had the opposite effect (Figure 6E,F). In vivo, AngII infusion elevated p‐PI3K, p‐Akt, p‐IκBα, and p‐p65 in murine abdominal aortas, changes that were reversed by CD5L silencing (Figure 6G). IHC confirmed these expression patterns (Figure 6H). Collectively, these data indicate that CD5L activates PI3K/Akt signaling to suppress NF‐κB activity, thereby limiting M1 macrophage polarization in the AAA microenvironment (Figure 6I).

### 3.6 Investigation of the mechanism by which CD5L reduces M1 macrophage polarization via the PI3K/Akt/NF‐κB axis

**FIGURE 7:**
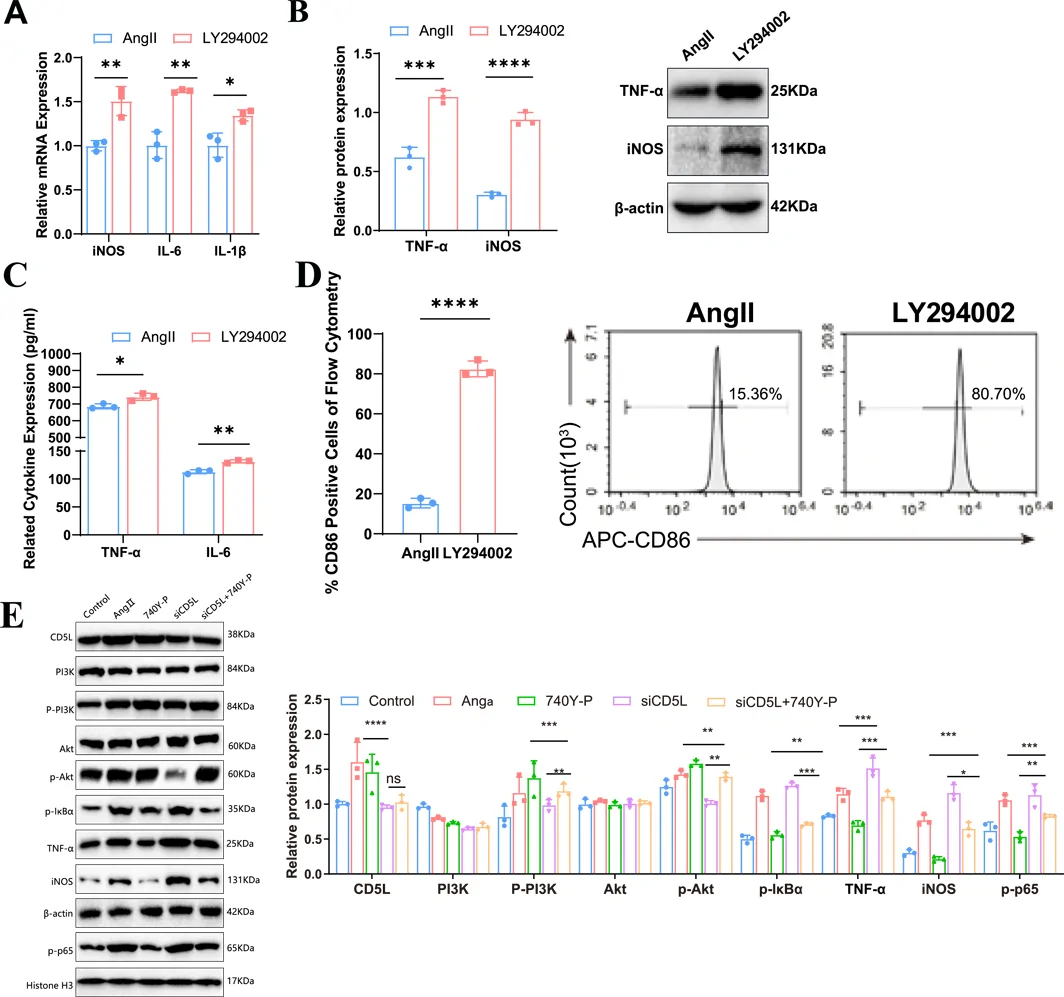
CD5L reduces M1 polarization by regulating the PI3K/Akt/NF‐κB axis. (A) qRT‐PCR results showing the gene expression levels of iNOS, IL‐6, and IL‐1β in RAW264.7 cells (*n* = 3). (B) Western blot results and densitometric analysis of TNF‐α and iNOS expression in RAW264.7 cells (*n* = 3). (C) ELISA analysis results of TNF‐α and IL‐6 levels in the supernatant of RAW264.7 cell cultures (*n* = 3). (D) Flow cytometry quantitative analysis and representative histograms identifying M1 macrophages (CD86+) in RAW264.7 cells (*n* = 3). (E) Western blot rescue experiment demonstrating that CD5L reduces M1 polarization in RAW264.7 cells by regulating the PI3K/Akt/NF‐κB axis (*n* = 3). Data are presented as mean ± SD. CD5L, cluster of differentiation 5‐like; ELISA, enzyme‐linked immunosorbent assay; qRT‐PCR, quantitative real‐time PCR. Statistical significance is indicated as *p < 0.05; **p < 0.01; ***p < 0.001; ****p < 0.0001; ns indicates no significant difference. [Correction added on 20 September 2026, after first online publication: The Figure 7 has been updated in this version.]

To further assess the role of PI3K/Akt signaling in CD5L‐mediated suppression of M1 polarization, we compared M1 markers between the AngII and LY294002 groups. qRT‐PCR, western blot analysis, and ELISA showed that the LY294002 treatment significantly increased iNOS, IL‐6, and IL‐1β expressions relative to AngII (Figure 7A–C). Flow cytometry confirmed an elevated proportion of CD86^+^ M1 macrophages in the LY294002 group (Figure 7D). A rescue experiment was then performed using the PI3K activator 740Y‐P (50 μg/mL; PMID: 31957758; PMID: 33961336) in control, AngII, 740Y‐P, siCD5L, and siCD5L + 740Y‐P groups. In siCD5L + 740Y‐P cells, CD5L expression was reduced compared to 740Y‐P alone, accompanied by decreased p‐PI3K and p‐Akt, increased p‐IκBα and nuclear p‐p65, and elevated TNF‐α and iNOS levels. Compared with siCD5L alone, 740Y‐P co‐treatment restored p‐PI3K and p‐Akt, suppressed p‐IκBα and p‐p65, and reduced TNF‐α and iNOS expression (Figure 7E). These findings support that CD5L attenuates M1 polarization in AAA through PI3K/Akt‐mediated inhibition of NF‐κB activity.

## 4 DISCUSSION

This study investigated the mechanism by which CD5L reduces macrophage M1 polarization in AAA through regulation of the PI3K/Akt/NF‐κB axis. AAA is a common and life‐threatening vascular disease characterized by localized dilation of the abdominal aortic wall accompanied by inflammatory cell infiltration and degenerative changes in the vascular wall.^11^, ^22^, ^23^ Despite its complex and incompletely elucidated pathogenesis, there are currently no effective pharmacological treatments available,^24^, ^25^ and surgical intervention remains the main therapeutic option. Therefore, identifying novel molecular targets is of great significance for early intervention and slowing AAA progression.

This study systematically revealed the regulatory role of CD5L in macrophage polarization during the formation and progression of AAA. By integrating public transcriptomic data and performing immune infiltration analysis, CD5L expression in AAA tissue was found to be significantly correlated with various immune cell subsets, showing a negative correlation with M1 macrophages. This suggests a role for CD5L in suppressing pro‐inflammatory macrophage polarization within the AAA microenvironment. In vitro experiments demonstrated that AngII stimulation simultaneously induced CD5L upregulation and enhanced M1 polarization; however, time‐course analysis revealed a peak shift between the two: CD5L expression continued to rise after stimulation, whereas M1 markers declined after 18 h, consistent with a negative feedback pattern during the later phase of inflammation.^8^, ^26^ Functional assays showed that CD5L knockdown markedly promoted M1 polarization of RAW264.7 cells and hMDMs, accompanied by elevated pro‐inflammatory cytokines and chemokines, whereas CD5L overexpression inhibited these changes. In ApoE^−/−^ mice with AAA, CD5L knockdown increased infiltration of M1 macrophages and upregulated pro‐inflammatory cytokine expression in the abdominal aorta. These findings are consistent with previous observations of CD5L‐mediated regulation of macrophage polarization in atherosclerosis models.^27^, ^28^

RNA‐seq and bioinformatics analyses indicated that CD5L suppresses M1 polarization in the AAA microenvironment through activation of the PI3K/Akt pathway. PI3K/Akt, a central regulator of cell survival, metabolism, and inflammation, is critically involved in cardiovascular pathology.^29^, ^30^ In AngII‐stimulated macrophages, the PI3K inhibitor LY294002, at concentrations that did not affect cell viability, significantly increased iNOS, IL‐6, and IL‐1β expressions as well as the proportion of CD86^+^ cells,^31^, ^32^ indicating that PI3K/Akt inhibition amplifies NF‐κB‐driven pro‐inflammatory responses. The PI3K activator 740Y‐P partially reversed the reductions in p‐PI3K/p‐Akt and the increase in NF‐κB signaling (p‐IκBα, p‐p65) induced by siCD5L while also lowering TNF‐α and iNOS levels, showing molecular and phenotypic effects opposite to LY294002. These results mechanistically establish PI3K/Akt as an upstream regulator of NF‐κB and as necessary for the anti‐inflammatory effect of CD5L.^33^ The results align with KEGG and GSEA enrichment analyses and are consistent with previous studies in other inflammatory models showing that PI3K/Akt activation restrains NF‐κB signaling and mitigates inflammation.^34^, ^35^

These results indicate that CD5L maintains or enhances PI3K/Akt activity, suppresses NF‐κB activation, reduces M1 polarization and pro‐inflammatory cytokine release, and thereby exerts a protective effect in the AAA microenvironment. This signaling axis provides a clear molecular target and pharmacological rationale for immune interventions aimed at modulating macrophage polarization. Clinically, CD5L represents a promising molecular target for early diagnosis and personalized treatment of AAA. High CD5L expression effectively suppresses inflammatory responses, supporting the development of interventions targeting M1 macrophage polarization to slow AAA progression and reduce the need for surgical intervention. Moreover, dynamic monitoring of CD5L expression levels could serve as a valuable biomarker for assessing AAA progression, offering a tool for early diagnosis and timely intervention.

Nevertheless, clinical translation of CD5L‐based interventions faces multiple challenges, including the lack of efficient and specific delivery systems, limitations in targeting precision, and concerns regarding long‐term safety and immunogenicity. Regulatory and drug development considerations also need to be addressed. Future work could explore nanoparticle‐, liposome‐, or viral vector‐based delivery platforms to improve CD5L stability and targeting in vivo, and conduct systematic pharmacokinetic, toxicological, and efficacy evaluations in large animal models prior to clinical application.

This study, while systematically elucidating the regulatory mechanism of CD5L in AAA, still has limitations. First, despite in vitro and in vivo validation of CD5L's function, the pathological process of AAA is highly complex, and single‐gene studies cannot fully capture its breadth. Second, the focus was primarily on M1 macrophages, without examining other relevant cell types such as T cells, neutrophils, and stromal cells. Third, the study relied mainly on the ApoE^−/−^ mouse model, which, although mimicking some features of human AAA, carries species‐specific differences and lacks validation in human clinical samples. Furthermore, although RNA‐seq suggested a role for CD5L in immunometabolic regulation, there was no direct experimental evidence from metabolomics or energy metabolism studies; the in vitro experiments were also limited to RAW264.7 murine macrophages without confirmation in primary human macrophages. It is worth noting that CD5L may exert dual or even opposing effects in diseases such as atherosclerosis, and the potential side effects of long‐term intervention—particularly on lipid metabolism—were not assessed.

Future studies should explore whether CD5L also regulates other immune and stromal populations in the AAA microenvironment, such as T cells, endothelial cells, and dendritic cells. Previous work has shown that cDC1s can promote CD8^+^ T‐cell recruitment and exacerbate AAA via an IRF8‐dependent pathway,^36^ suggesting that CD5L might act within a broader immune network. Multi‐omics approaches, including transcriptomics, metabolomics, and proteomics, should be integrated to map the global regulatory landscape of CD5L in immunometabolism and vascular inflammation. Validation of the PI3K/Akt‐NF‐κB axis in primary human macrophages and AAA patient samples is warranted, as is assessment of CD5L's functional diversity across different pathological contexts. At the pharmacological level, given the systemic toxicity of PI3K inhibitors such as LY294002, strategies such as liposome‐ or antibody‐conjugated delivery, or the development of more selective and less toxic PI3K/Akt modulators, should be pursued. It is worth noting that local administration of LY294002 (e.g., intraocular injection) has been shown to effectively inhibit pathological angiogenesis without inducing systemic side effects or impairing function.^37^ In addition, long‐term systemic administration in rat models (1.2 mg/kg/day for 4 weeks) improved cardiac function without significantly affecting blood pressure or glucose levels.^38^ Encapsulation of LY294002 within polymer nanoparticles has also been reported as a feasible targeted delivery strategy to reduce systemic exposure and toxicity risk.^39^

## 5 CONCLUSION

CD5L was found to be upregulated in AAA and acted as a negative regulator of M1 macrophage polarization through activation of the PI3K/Akt/NF‐κB signaling axis. This modulation reduced pro‐inflammatory cytokine release and vascular injury, thereby mitigating AAA progression. Both in vitro and in vivo evidence indicated that the PI3K/Akt pathway was essential for the anti‐inflammatory effects of CD5L, with pharmacological activation partially restoring the protective phenotype under CD5L deficiency. These findings suggest that enhancing CD5L activity or selectively activating the PI3K/Akt pathway in macrophages could represent a rational approach for controlling vascular inflammation and slowing AAA development. Translation into clinical applications will require optimization of delivery systems, comprehensive safety assessment, and validation in human samples (Graphic abstract).

## AUTHOR CONTRIBUTIONS

H.Y., N.L., and Z.W. performed experiments, data acquisition, and analysis. L.L. and T.L. contributed to bioinformatics analyses and interpretation of RNA sequencing datasets. Q.L. provided technical support for in vivo studies. M.D. and T.W. conceived and supervised the project, designed the research, and secured funding. H.Y. drafted the manuscript with input from all authors. All authors critically reviewed the manuscript and approved the final version.

## CONFLICT OF INTEREST STATEMENT

The authors declare no conflicts of interest.

## ETHICS STATEMENT

All animal experiments were approved by the Animal Ethics Committee of the Harbin Scientia Biotechnology Corporation (No. HRBSCIEC20230310).

## CONSENT FOR PUBLICATION

Not applicable.

## Supporting information

Figure S1

## Data Availability

All data supporting the findings of this study are available within the paper and its Supplementary Information. Additional data can be requested from the corresponding author.
