# Correction to “Targeting the PI3K/Akt/NF‐κB axis: Cluster of differentiation 5‐like‐mediated immunometabolic regulation of macrophage polarization in abdominal aortic aneurysm”

**DOI:** 10.1002/ccs3.70113

**Published:** 2026-09-22

Yi, Hemoren, Nan Liu, Zhengyang Wu, et al. 2025. “Targeting the PI3K/Akt/NF‐κB Axis: Cluster of Differentiation 5‐Like‐Mediated Immunometabolic Regulation of Macrophage Polarization in Abdominal Aortic Aneurysm.” *Journal of Cell Communication and Signaling*: e70048. https://doi.org/10.1002/ccs3.70048.

In the originally published article, the authors identified an inconsistency in the presentation of the control group in Figure 2. The original results for the control group did not align with the statistical logic required by the experimental design. Upon verification, the authors confirmed that this arose from an incorrect application of the control group definition.

As described in the Methods section, the “control Ang II‐untreated group” refers to the baseline control group without Ang II treatment. To ensure that the experimental groups and results are consistent in terms of statistical logic, the control group in Figure 2 has been corrected to represent the baseline state without Ang II intervention.

Additionally, Figures 3, 6, and 7 have been updated to correct typos in the figure labeling. The corrected Figures 2, 3, 6 and 7 are shown below.

The funding source has been corrected to “Health Science and Technology Capacity Improvement Project of Jilin Province (2022LC035).”

We apologize for these errors.


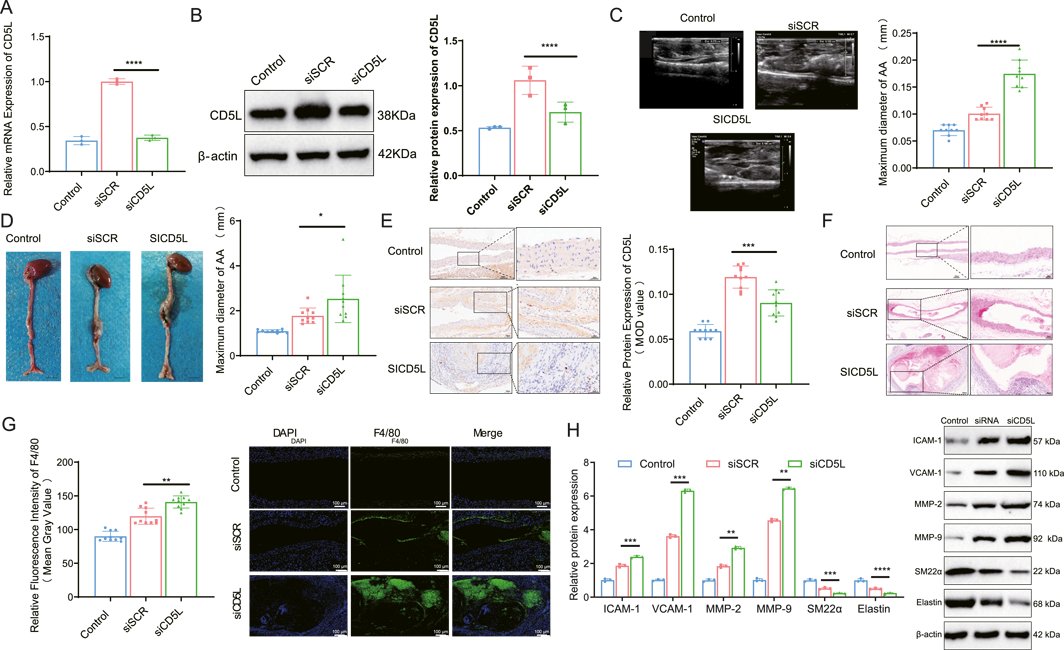



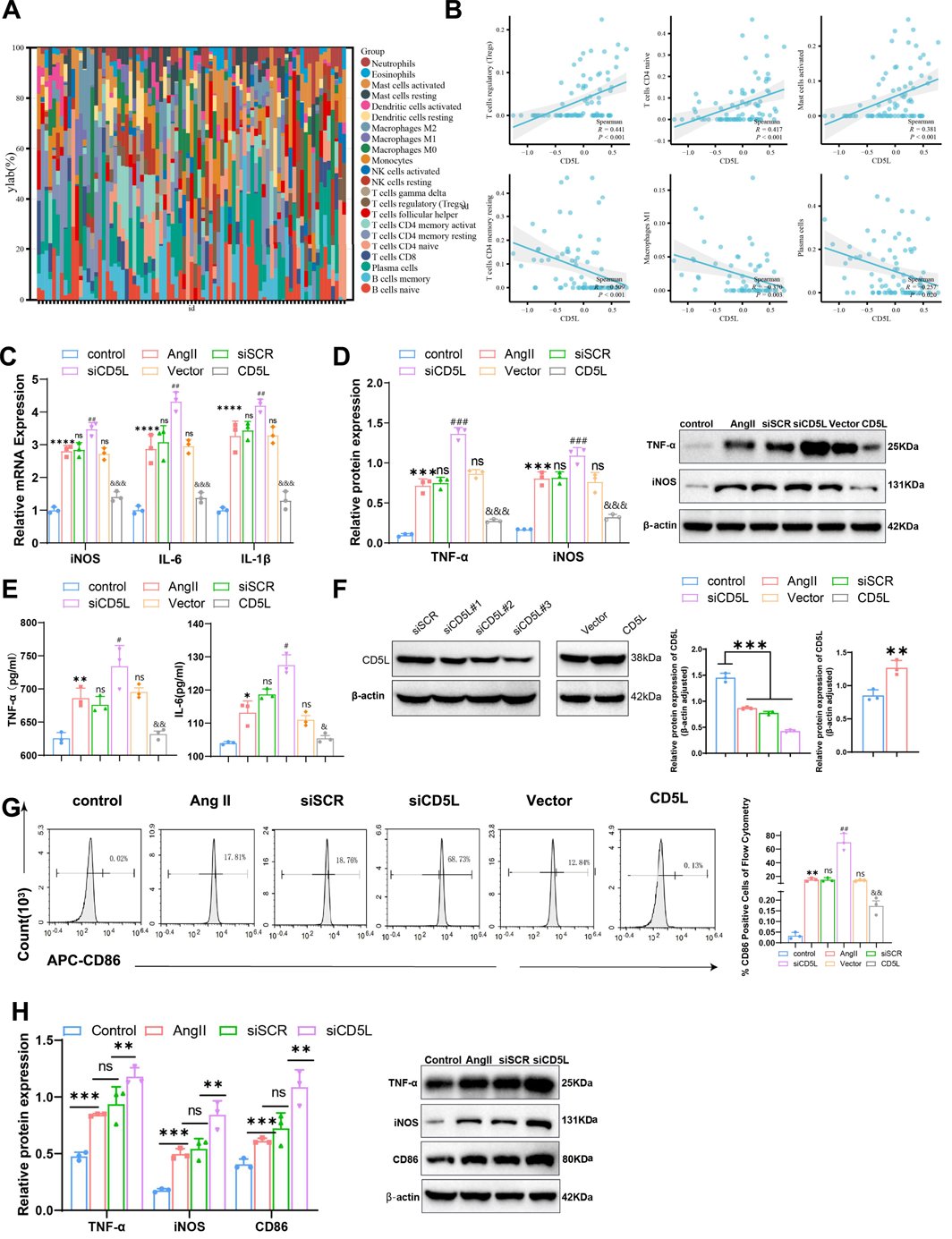



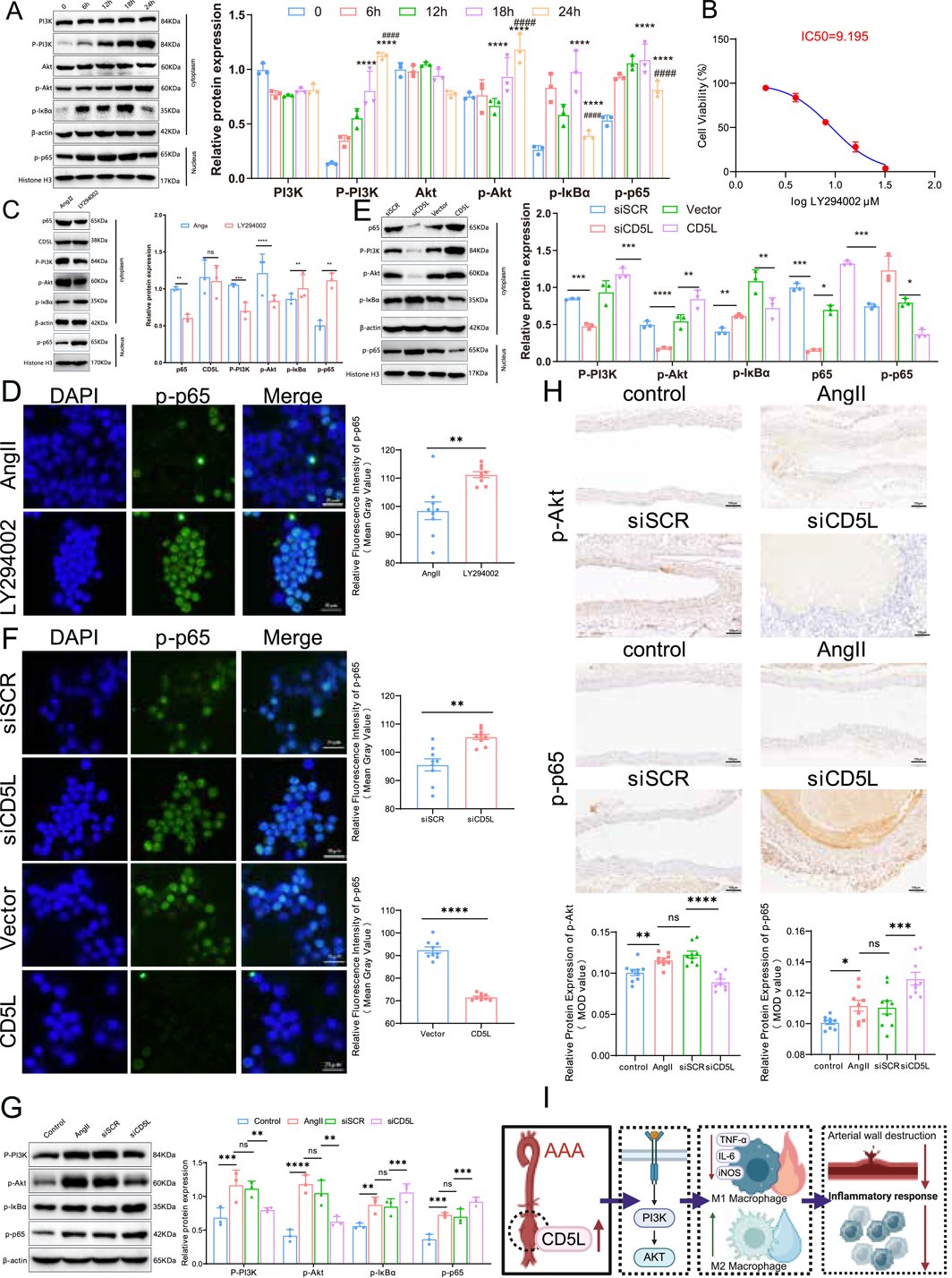



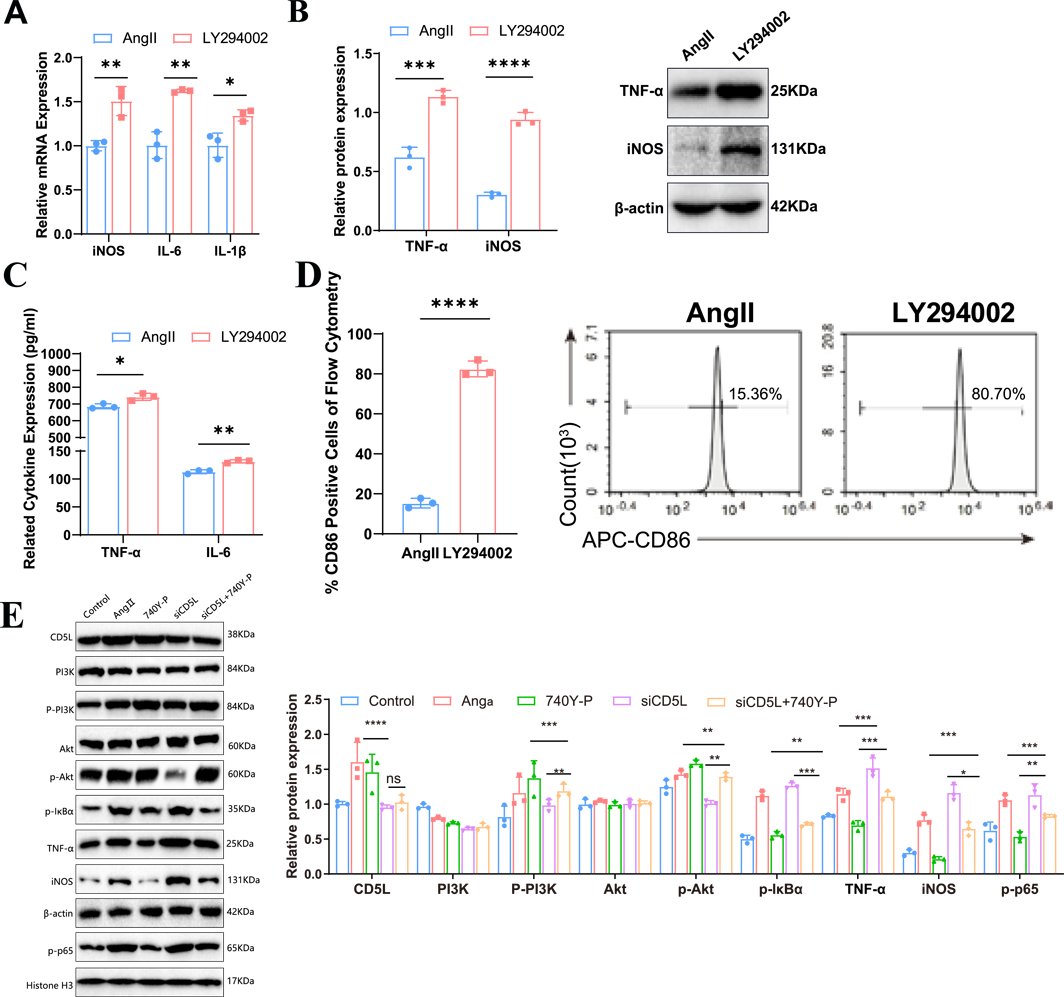


## Supporting information


Figure S1

