# Supplementary material for: Correction to “Targeting the PI3K/Akt/NF‐κB axis: Cluster of differentiation 5‐like‐mediated immunometabolic regulation of macrophage polarization in abdominal aortic aneurysm”
Source: J Cell Commun Signal. 2026 Sep 22;20(3):e70113. doi: 10.1002/ccs3.70113 (PMC13595395; doi:10.1002/ccs3.70113)
Supplement: Supplementary file 1 [file CCS3-20-e70113-s001.docx]

**Figure S1. Effects of CD5L on M1 polarization of hMDMs and mitochondrial energy metabolism in the AAA condition.**

Note: (A) Flow cytometry-based quantitative analysis confirming CD86 and CD206 expression in RAW264.7 cells under resting conditions (n = 3). (B) qRT-PCR analysis of iNOS, IL-6, and IL-1β gene expression in hMDMs (n = 3). (C) Western blot analysis and densitometric quantification of TNF-α and iNOS expression in hMDMs (n = 3). (D) ELISA analysis of TNF-α and IL-6 levels in the supernatants of hMDM cultures (n = 3). (E) Western blot analysis and densitometric quantification of CD5L knockdown and overexpression in hMDMs (n = 3). (F) Seahorse XF Cell Mito Stress assay assessing mitochondrial energy metabolism, including basal respiration, maximal respiration, and ATP production (n = 3). Data are presented as mean ± s.d. Statistical significance: **p <* 0.05, ***p <* 0.01, ****p <* 0.001, *****p <* 0.0001 vs. control; #*p <* 0.05, ##*p <* 0.01, ###*p <* 0.001, ####*p <* 0.0001 vs. siSCR; &*p <* 0.05, &&*p <* 0.01, &&&*p <* 0.001, &&&&*p <* 0.0001 vs. Vector; ns, not significant vs. AngII.
